# In Situ Multi-Scale Characterization of Tensile Damage Evolution in Low-Braiding-Angle 3D Braided CFRP Composites for Propeller Blades

**DOI:** 10.3390/ma19101982

**Published:** 2026-05-11

**Authors:** Zhihua Zhang, Fangcheng Zheng, Guohua Fan, Mingming Xu

**Affiliations:** 1 Key Laboratory for Light-Weight Materials, Nanjing Tech University, Nanjing 211816, China; 202361103060@njut.edu.cn (Z.Z.); zhengfc@mat-jitri.cn (F.Z.); 2 Marine Science and Technology Domain, Beijing Institute of Technology, Zhuhai 519088, China

**Keywords:** braiding composites, in situ X-ray CT, digital volume correlation

## Abstract

Three-dimensional braided carbon-fiber-reinforced polymer (CFRP) composites are promising for lightweight aircraft propeller blades. Aircraft composite structures may approach temperatures of 80–90 °C under the combined effects of solar radiation, infrared heating, and ground reflection. Yet the thermo-mechanical failure mechanisms of low-braiding-angle architecture remain insufficiently understood. This study comparatively investigates the tensile behavior and damage evolution of low-angle four-directional (3D4A-20°) and five-directional (3D5A-20°) braided CFRP composites under axial tension at both room temperature and 90 °C. A multi-scale approach integrating in situ X-ray computed tomography, digital image correlation, digital volume correlation, and scanning electron microscopy was used to characterize strain localization, internal cracking, and fracture morphology. At room temperature, 3D5A-20° shows higher stiffness and strength than 3D4A-20° because additional axial yarns improve load-transfer and three-dimensional constraint. At 90 °C, matrix softening and interfacial degradation accelerate crack initiation, strain localization, and damage propagation in both architectures. Nevertheless, 3D5A-20° maintains more stable and progressive damage evolution, whereas 3D4A-20° exhibits earlier crack coalescence and greater mechanical degradation. Overall, elevated temperature accelerates damage evolution through matrix softening and interfacial degradation, whereas braided architecture determines load transfer and crack connectivity. These findings provide guidance for the design of low-angle braided composites for thermally exposed aircraft propeller blades.

## 1. Introduction

With the increasing replacement of metallic materials by composite systems in aerospace propeller and fan blades, 3D braided carbon fiber reinforced polymer (CFRP) composites have attracted considerable attention because of their low density, high axial tensile strength, and good structural integrity [1,2,3,4]. In particular, low-braiding-angle architectures are promising candidates for blade-like load-bearing components, since a smaller braiding angle generally improves fiber alignment along the loading direction and thus enhances tensile load-carrying efficiency [5,6,7]. However, the service conditions of such components are complex [8]. During operation, blades are subjected to high-speed rotational centrifugal tension, repeated loading-unloading cycles, aerodynamic loads, and environmental fluctuations such as temperature rise and hygrothermal exposure [8,9,10,11]. Although propeller blades are subjected to multiple complex loading conditions during service, including bending, torsion, and aerodynamic loads, the spanwise tensile load induced by rotation remains one of their key loading components [12,13,14]. With the continuous increase in bypass ratio and blade length in advanced propulsion systems, the tensile load sustained by blade structures becomes more severe, placing higher demands on the strength, durability, and damage tolerance of braided composites. In addition, the thermal environment of aircraft composite structures can be significantly intensified by direct solar radiation, infrared radiation, and reflections from the ground/tarmac, as indicated by FAA thermal modeling and composite-structure qualification guidance [15]. Since spanwise tensile loading is one of the key service loads of propeller blades and thermally exposed composite blade structures can reach approximately 80–90 °C, axial tensile testing at 90 °C was adopted in this study as a representative thermo-mechanical loading condition to evaluate the mechanical properties and damage evolution of low-braiding-angle 3D braided composites [16,17].

In recent years, extensive investigations have been conducted widely on 3D braided composites from the macro-scale mechanical response to the micro-scale damage and failure mechanisms. Existing studies have systematically examined their tensile behavior, damage evolution, and structural characteristics through experimental characterization, numerical simulation, and multi-scale analysis. These efforts have provided an important foundation for understanding the mechanical performance and failure behavior of 3D braided composites under different loading and environmental conditions. In terms of macroscopic tensile behavior, many researchers have carried out detailed investigations. Fang [18] carried out uniaxial tensile tests to investigate the longitudinal stress-strain response and fracture morphologies of three-dimensional four-directional braided composites with different specimen thicknesses. Zhou [19] indicated that the 3D four-directional braided composites exhibited slight nonlinearity and relatively large failure strain under uniaxial tension, and that the tensile fracture process was governed by the coupled effects of fiber fracture, matrix cracking, and interfacial debonding. Through transverse uniaxial tensile tests on three-dimensional five-directional braided composites, the authors concluded that these composites exhibit a pronounced stage-wise damage evolution under transverse tension, with the load-displacement curve gradually transitioning from an initial linear response to damage accumulation and finally to sudden failure, while surface cracks rapidly initiate and propagate as the applied load approaches the ultimate level. Wan [20] showed, by combining moisture-aging tests, static tensile tests, tension-tension fatigue tests, and micro-CT characterization, that hygrothermal aging not only significantly degrades the static and fatigue properties of carbon/glass 3D five-directional hybrid braided composites, but also alters the damage-initiation sites, crack-propagation paths, and final failure modes.

At the micro-scale, based on an experimentally validated multi-scale finite element model, Zhang [21] showed that forming-induced damage in 3D braided preforms is mainly concentrated in the interlaced region and is jointly governed by shear stress, inter-fiber friction, and beating-up height. By combining tensile experiments with continuum-damage-mechanics-based finite element analysis, Zhang [22] investigated the longitudinal tensile behavior of 3D four-directional, five-directional, and six-directional braided composites, and showed that the braiding architecture and yarn configuration significantly affect the tensile strength, modulus, crack-propagation path, and final failure mode of the composites. Ya [23] employed X-ray computed tomography (CT) as a non-destructive characterization method and introduced glass-fiber tracers to enhance contrast; combined with 3D reconstruction and segmentation.

This approach enabled quantitative extraction of yarn trajectories/cross-sectional geometries and the three-dimensional distribution, morphology, and content of porosity in 3D5A braided composites. Li et al. [24] reconstructed realistic yarn cross-sections using Micro-CT and established a parametric finite-element progressive-damage prediction framework for 3DR5d three-dimensional rotational five-directional braided composites. Many researchers have also systematically investigated the effects of braiding angle and fiber volume fraction on tensile mechanical properties and damage mechanisms [25,26,27,28].

Existing studies have improved the understanding of the macroscopic response and individual damage mechanisms of 3D braided composites. However, previous studies have mainly focused on either experimental mechanical testing or isolated microscopic characterization, while systematic investigations integrating multi-scale damage evolution remain limited. This limitation is particularly evident for low-braiding-angle 3D braided composites, which are highly relevant to blade-like load-bearing components such as aerospace propeller and fan blades, yet remain insufficiently characterized in terms of tensile damage initiation, crack evolution, and final fracture mechanisms. Therefore, the present study focuses on low-braiding-angle braided composites suitable for blade applications and aims to clarify the effects of braided architecture and temperature on their tensile behavior, strain localization, crack propagation, and fracture mechanisms through a multi-scale experimental framework. In particular, under the combined effects of braided architecture and elevated temperature, the full process of crack evolution from initiation to propagation and final coalescence has not yet been clarified using direct and quantitative multi-scale evidence.

To address this issue, the present study systematically compares the tensile behaviors of 3D four-directional (3D4A) and 3D five-directional (3D5A) braided composites with the same braiding angle of 20° at room temperature and 90 °C. By integrating stress-strain analysis, full-field strain measurements by digital image correlation (DIC) and digital volume correlation (DVC), CT and high-temperature in situ CT, and fracture-surface observation, a multi-scale experimental framework is established to capture the complete damage-evolution process. The results demonstrate that temperature primarily accelerates damage evolution by softening the matrix and weakening the fiber-matrix interface, whereas braided architecture governs the sensitivity to thermally induced damage through its regulation of load-transfer pathways and three-dimensional constraint. These findings provide direct experimental evidence for understanding and optimizing the thermo-mechanical durability of low-braiding-angle 3D braided composites.

## 2. Materials and Methods

### 2.1. Fabrication of 3D Braided Preforms

**Figure 1:**
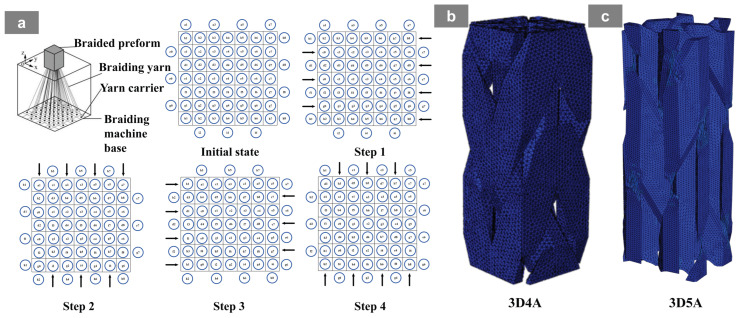
Schematic illustration of the four-step braiding process and mesoscopic models of 3D braided composites: (**a**) braiding principle and yarn-carrier movement sequence in the four-step braiding method; (**b**) mesoscopic model of the 3D4A braided architecture; (**c**) mesoscopic model of the 3D5A braided architecture.

The 3D braided composite preforms used in this study were reinforced with T300-3K carbon fiber tows supplied by Toray. As illustrated in Figure 1a, the preforms were fabricated using the conventional four-step braiding process, in which one complete braiding cycle consists of four successive yarn-carrier movements with a unit step in each stage. Specifically, adjacent yarn rows are alternately shifted in the x-direction in Step 1, adjacent yarn columns are alternately shifted in the y-direction in Step 2, and Steps 3 and 4 reverse the motions of Steps 1 and 2, respectively. After each braiding cycle, a beating-up operation is performed to densify the preform and maintain structural stability, while the periodic length along the z-direction is defined as the braiding pitch length h. Repetition of this four-step sequence produces the required 3D four-axial braided preform [29]. Based on this process, the 3D five-axial architecture was obtained by introducing axial yarn carriers between adjacent braiding yarn carriers in each row or column, thereby enhancing the longitudinal load-bearing capability of the preform. The braided architectures of 3D4A and 3D5A are shown in Figure 1b and c, respectively [30].

### 2.2. Fabrication of 3D Braided CFRP Composites

**Figure 2:**
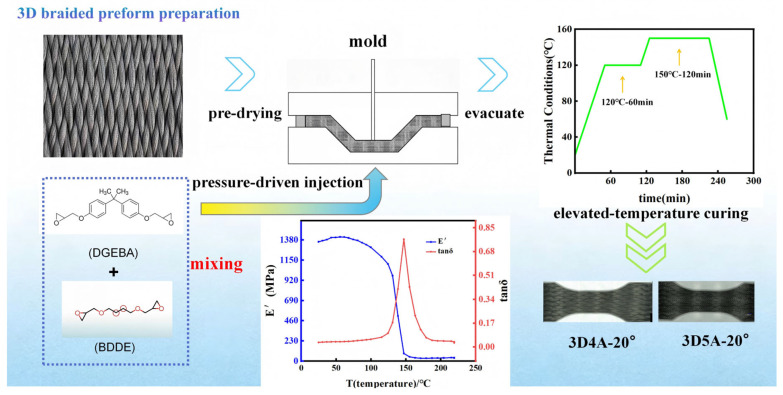
Schematic illustration of the fabrication process for the 3D braided CFRP composites.

The fabrication of the 3D braided CFRP composites involved fiber braiding, resin impregnation, vacuum curing, and post-processing, as schematically illustrated in Figure 2. T300-3K carbon fiber tows were employed to manufacture the designed textile architecture via a three-dimensional braiding process. The braided preforms were then impregnated with a mixed epoxy resin system consisting of diglycidyl ether of bisphenol A (DGEBA) and 1,4-butanediol diglycidyl ether (BDDE), together with the curing agent methyl hexahydrophthalic anhydride and the accelerator 2,4,6-tris(dimethylaminomethyl) phenol, to ensure thorough infiltration of the resin into the fiber tows. Subsequently, the impregnated preforms were placed into a mold and vacuum-cured. According to the DMA curve, the glass transition temperature (Tg) of the matrix is 147 °C. The curing schedule was as follows: starting from room temperature, the temperature was ramped to 120 °C and held for 60 min under a vacuum pressure of −100 kPa to ensure sufficient resin flow and impregnation; the temperature was then increased to 150 °C, which is close to the Tg of the matrix, and held for 120 min under −100 kPa to promote further crosslinking and enhance the mechanical performance of the composite; finally, the laminate was cooled to 60 °C. As shown in Figure 2, the heating rate and cooling rate were 2 °C/min and 3 °C/min, respectively. This curing process promoted effective fiber-matrix consolidation, yielding composites with improved mechanical performance and durability [31]. The structural parameters of the fabricated 3D braided composites are summarized in Table 1. The mechanical properties of the matrix and T300-3K carbon fiber are given in Table 2.

### 2.3. Specimen Description (Braiding Angle and Dimensions)

**Figure 3:**
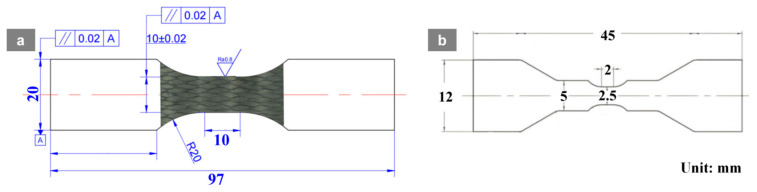
(**a**) Dimensions of the macroscopic tensile specimen; (**b**) dimensions of the in situ CT tensile specimen.

Tensile specimens were prepared in accordance with the standard for determining the tensile-impact strength of plastics (ISO 8256:2004) [32]. Figure 3a shows a dog-bone tensile specimen with an overall length of 97 mm, a gauge-section width of 10 ± 0.02 mm and a gauge length of 10 mm, a transition fillet radius of 20 mm, and a surface roughness of Ra 0.8 μm; a geometric tolerance of 0.02 mm is specified with respect to datum A. Figure 3b shows a miniature in situ CT tensile specimen with an overall length of 45 mm, an end width of 12 mm, a reduced-section width of 5 mm, a minimum feature size of 2 mm, and a notch-root fillet feature size of 2.5 mm. All dimensions are given in millimeters. The specimens were cut from the fabricated laminates using laser cutting followed by CNC precision machining [33,34].

### 2.4. Tensile Testing System

**Figure 4:**
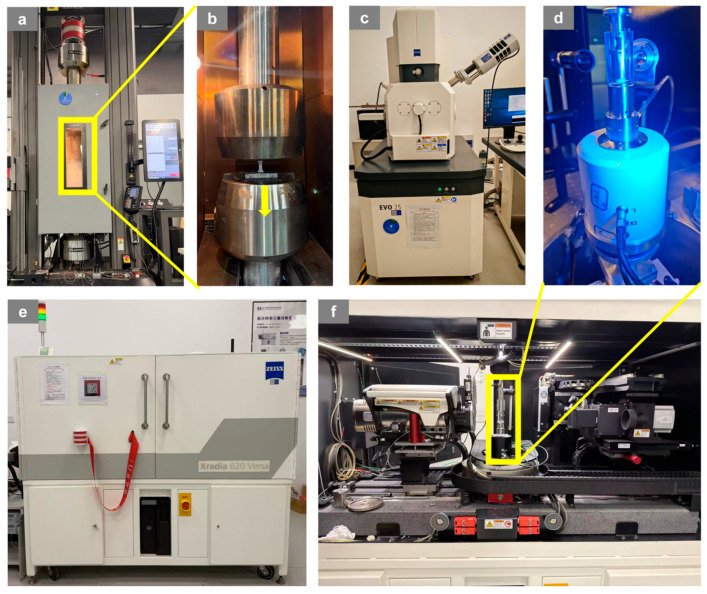
(**a**) High-temperature furnace tensile testing machine; (**b**) tensile testing setup; (**c**) tungsten-filament scanning electron microscope; (**d**) in situ tensile stage; (**e**) ZEISS Xradia 620 Versa X-ray micro-computed tomography system; (**f**) X-ray source and detector.

Tensile tests were carried out on a universal testing machine (Instron 5985, Instron, Norwood, MA, USA), as shown in Figure 4a. The dimensions of the tensile specimen are shown in Figure 4a. The system was equipped with a high-stiffness dual-column load frame, a high-accuracy load cell, and closed-loop displacement/strain control, providing stable quasi-static loading with a maximum capacity of 250 kN [35]. Specimens were axially clamped using wedge grips, and for elevated-temperature tests, a furnace with an operating range of 25–350 °C was integrated into the testing system, as shown in Figure 4b. The target temperature was set to 90 °C with a heating rate of 3 °C/min, followed by a 30 min dwell to ensure thermal uniformity prior to loading. The loading rate was fixed at 1 μm/s, corresponding to a nominal strain rate is 1 × 10^−4^ s^−1^. Three samples per material were tested to avoid stochastic effects, and the relative errors were all below 5%, indicating that the measured data are accurate. The DIC analysis was performed using VIC-2D 6 software (Correlated Solutions, Irmo, SC, USA). A random black speckle pattern was sprayed on the specimen surface prior to testing. The acquired image resolution was 460 × 1120 pixels. For image correlation, a subset size of 21 × 21 pixels and a step size of 5 pixels were adopted, which are sufficient to capture the strain localization behavior while maintaining computational stability. The axial strain field was calculated from the full-field displacement gradients obtained by the correlation algorithm. The DIC measurement uncertainty was evaluated based on repeated unloaded image pairs, and the observed localization bands were significantly greater than the noise floor. Fracture morphologies and microscopic damage features of the specimens were further examined using a TESCAN VEGA3 XMH tungsten-filament scanning electron microscope (SEM) as shown in Figure 4c, which enabled clear characterization of microscopic failure features such as fiber fracture, interfacial debonding, matrix cracking, and fiber pull-out. In addition, X-ray CT experiments were conducted using a ZEISS Xradia 620 Versa high-resolution three-dimensional imaging system, as shown in Figure 4e,f. The dimensions of the in situ CT specimen are shown in Figure 3b, combined with a WK-XFunct in situ loading stage installed inside the CT chamber, as shown in Figure 4d. The loading stage provided a maximum tensile/compressive load of 5 kN and operated over a temperature range of −20 °C to 1000 °C. CT scans were acquired at an accelerating voltage of 70 kV and a tube power of 8.5 W, with a voxel size of 5.12 μm, an exposure time of 1 s per projection, and 1600 projections per scan, ensuring sufficient signal-to-noise ratio and high-quality three-dimensional reconstructions for subsequent image processing and quantitative analysis [36]. Figure 3b shows the dimensions of the in situ CT tensile specimen and the macroscopic tensile specimen. The CT scan data was processed in Avizo2022 for crack extraction through three-dimensional reconstruction of stacked 2D slices, region-of-interest selection, median filtering and contrast enhancement, followed by consistent global threshold segmentation and subsequent crack visualization using volume and surface rendering at different loading stages. DVC analysis was performed on the reconstructed CT volumes with a voxel size of 5.12 µm. A correlation cell size of 400 µm and an overlap of 50% were used.

## 3. Results and Discussion

### 3.1. Stress-Strain Analysis

**Figure 5:**
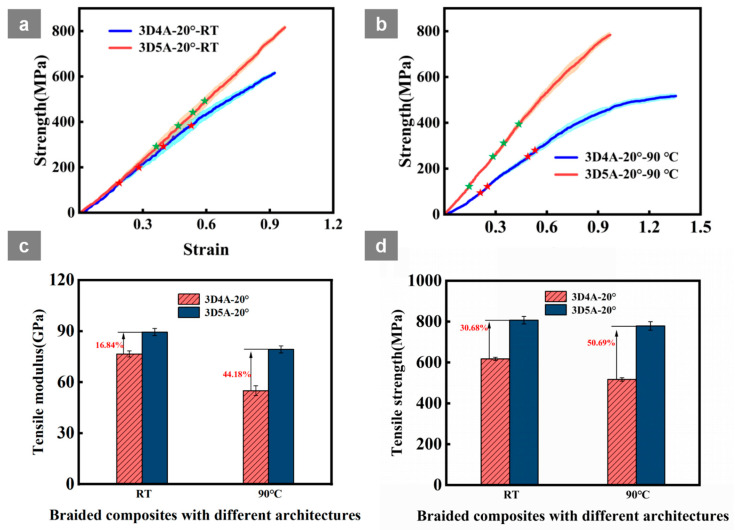
Tensile behavior and tensile properties of the 3D4A-20° and 3D5A-20° braided composites at room temperature (RT) and 90 °C: (**a**) tensile stress-strain curves at RT; (**b**) tensile stress-strain curves at 90 °C; (**c**) comparison of tensile modulus; (**d**) comparison of tensile strength.

Figure 5a,b presents the tensile stress-strain responses of the 3D braided CFRP specimens at room temperature (RT) and 90 °C, while the corresponding tensile modulus and tensile strength are summarized in Table 3 and further compared in Figure 5c,d. Values are reported as mean ± standard deviation (*n* = 3). Two low-braiding-angle architectures were compared, namely the four-directional composite (3D4A-20°) and the five-directional composite (3D5A-20°), in order to clarify the role of the additional axial yarn system in thermo-mechanical performance. The marked points on the curves indicate the load levels selected for the in-situ tensile tests.

At RT, 3D5A-20° exhibits a steeper initial slope and a higher peak stress than 3D4A-20°, indicating superior stiffness and tensile strength. The tensile modulus increases from 76.6 GPa to 89.5 GPa, while the tensile strength rises from 617.6 MPa to 807.1 MPa. This improvement is attributed to the additional axial yarns, which enhance load-transfer efficiency and structural redundancy, thereby suppressing early localization.

Both composites show reduced stiffness and strength because of matrix softening and interfacial weakening, but the extent of degradation is strongly architecture-dependent. For 3D4A-20°, the tensile modulus decreases to 55.0 GPa and the tensile strength decreases to 516.9 MPa, corresponding to reductions of 28.20% and 16.31%, respectively. By contrast, 3D5A-20° retains a much larger fraction of its RT properties, with the modulus decreasing to 79.3 GPa and the strength to 778.9 MPa, corresponding to reductions of 2.42% and 10.25%, respectively. The 3D5A-20° curves therefore remain steeper and more stable at elevated temperature, indicating that the added axial yarn system improves three-dimensional constraint and load sharing, thereby alleviating temperature-induced localization and delaying crack linkage.

Overall, the results at 90 °C demonstrate that 3D5A-20° maintains higher tensile modulus and strength than 3D4A-20° under both room-temperature and elevated-temperature conditions. Owing to its improved load-transfer efficiency, higher structural redundancy, and better resistance to temperature-induced performance degradation, the 3D5A architecture is more advantageous for blade-like structures operating under tensile-dominated loading conditions.

### 3.2. Surface Crack Propagation

**Figure 6:**
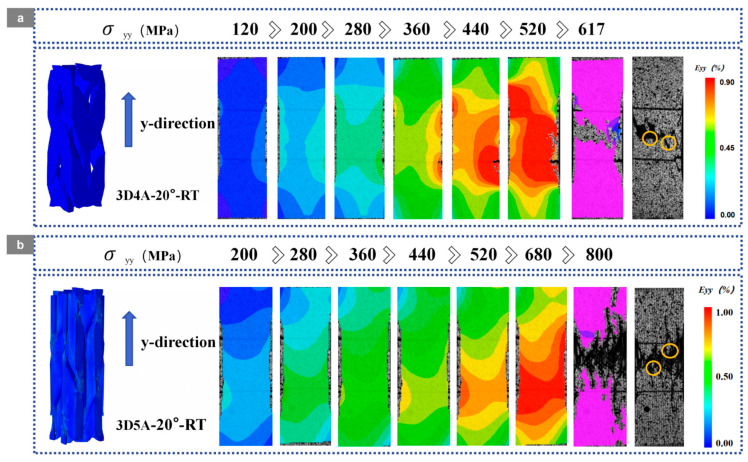
DIC strain contour maps of the 3D braided composites with a braiding angle of 20° at room temperature (RT): (**a**) 3D4A-20°; (**b**) 3D5A-20°.

**Figure 7:**
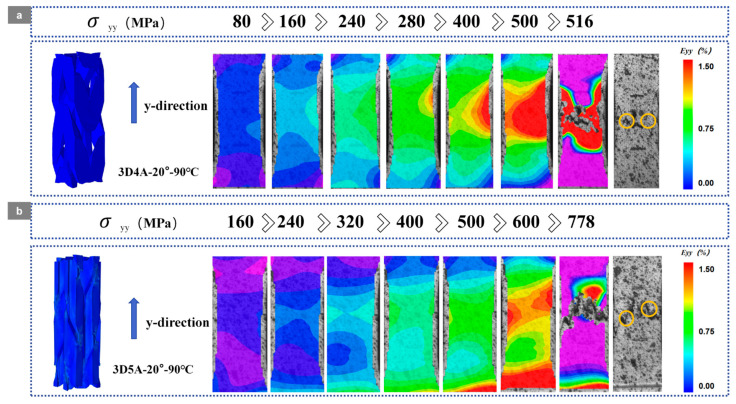
DIC strain contour maps of the 3D braided composites with different axial architectures at a braiding angle of 20° under elevated temperature (90 °C): (**a**) 3D4A-20°; (**b**) 3D5A-20°.

Based on the DIC full-field axial strain E_yy_ maps shown in Figure 6 and Figure 7, the damage evolution of the 20° braided composites is strongly temperature dependent, while the axial architecture controls the localization pattern and the associated temperature sensitivity. The regions indicated on the fracture diagrams in Figure 6 and Figure 7 were further examined by SEM for detailed analysis.

As shown in Figure 6 and Figure 7, under room-temperature conditions, the 3D4A-20° and 3D5A-20° composites exhibit distinct strain-localization behaviors. For 3D4A-20°, the strain field remains relatively uniform at low stress and gradually concentrates with increasing load. At the late stage, a dominant high-strain band develops and intensifies, followed by rapid localization and final fracture. In contrast, 3D5A-20° exhibits a more stable and spatially distributed strain evolution over a wider stress range. Although local high-strain regions gradually emerge, pronounced localization is mainly confined to the final loading stage. Compared with 3D4A-20°, the 3D5A-20° architecture maintains a more distributed deformation pattern, indicating more effective load sharing and stronger three-dimensional constraint under room-temperature loading.

At 90 °C, strain localization is promoted in both architectures and occurs earlier than at room temperature. For 3D4A-20°, pronounced localized regions appear at lower stress levels and intensify rapidly with further loading. The final stage is characterized by severe strain concentration together with an accelerated transition to crack growth and failure, which is consistent with thermally induced matrix softening and interfacial degradation. By comparison, 3D5A-20° also shows earlier localization at elevated temperature, but the strain field remains comparatively more uniform up to higher stress levels, and strong localization is still mainly concentrated near the final stage. Relative to 3D4A-20°, the 3D5A-20° composite retains a more distributed strain pattern at elevated temperature, suggesting that the additional axial yarn system improves load redistribution and delays the formation of a single dominant localization band.

Accordingly, representative macroscopic tensile fracture morphologies were selected for further analysis in order to correlate the strain-localization behavior with the final failure characteristics of the composites.

### 3.3. Fracture-Surface Morphology Analysis

SEM fractography further confirms that temperature is the dominant factor accelerating damage in the low-braiding-angle composites, while the axial architecture, changing from the four-axial to the five-axial system, controls how this thermal degradation is expressed at the fracture surface.

**Figure 8:**
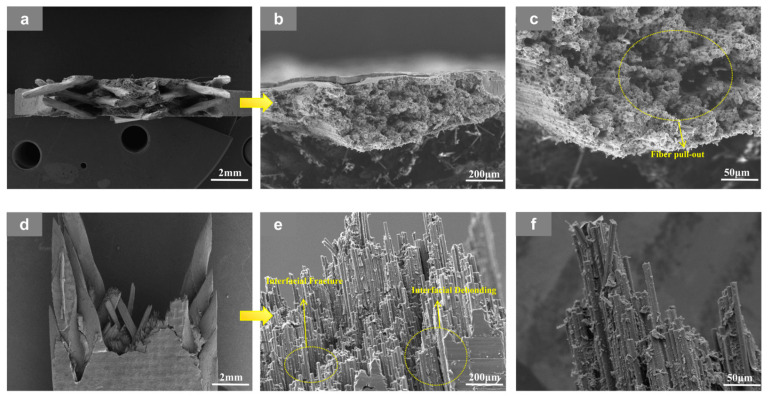
SEM images of the fracture surface of the 3D4A-20° specimen after tensile testing at room temperature (RT), shown at different magnifications: (**a**) top views at 20×; (**b**) top views at 200×; (**c**) top views at 1000×; (**d**) front views at 20×; (**e**) front views at 200×, and (**f**) front views at 1000×.

**Figure 9:**
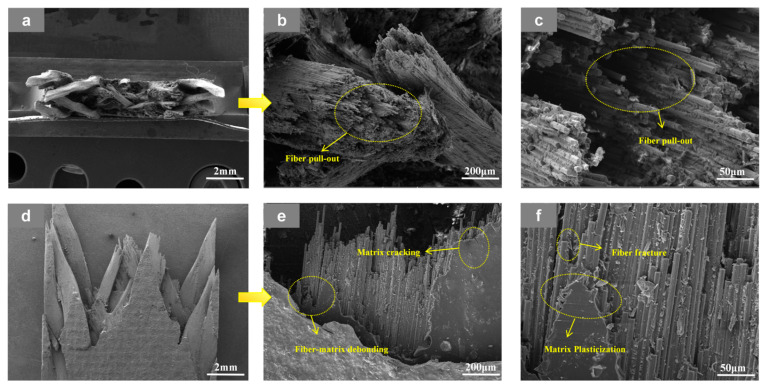
SEM images of the fracture surface of the 3D4A-20° specimen after tensile testing at 90 °C at room temperature (RT), shown at different magnifications: (**a**) top views at 20×; (**b**) top views at 200×; (**c**) top views at 1000×; (**d**) front views at 20×; (**e**) front views at 200×, and (**f**) front views at 1000×.

At RT, the 3D4A-20° specimens in Figure 8 exhibit a fracture mode dominated by interfacial debonding and fiber-controlled failure. Clear interfacial separation, interfacial fracture, fiber pull-out, and local fiber fracture can be observed, indicating that damage initiates at the fiber-matrix interface and then develops along the concentrated yarn-dominated load path. After testing at 90 °C in Figure 9, the same architecture shows more severe fiber pull-out, enlarged fiber-matrix debonding, obvious matrix cracking, and local matrix plastic deformation. These features indicate that matrix softening and interfacial weakening at elevated temperature reduce the resistance to crack growth and promote a faster transition from local debonding to unstable fracture. In other words, the four-axial architecture is more sensitive to temperature because its load-transfer is concentrated in fewer dominant yarn channels, so thermal degradation is more readily converted into rapid damage accumulation.

**Figure 10:**
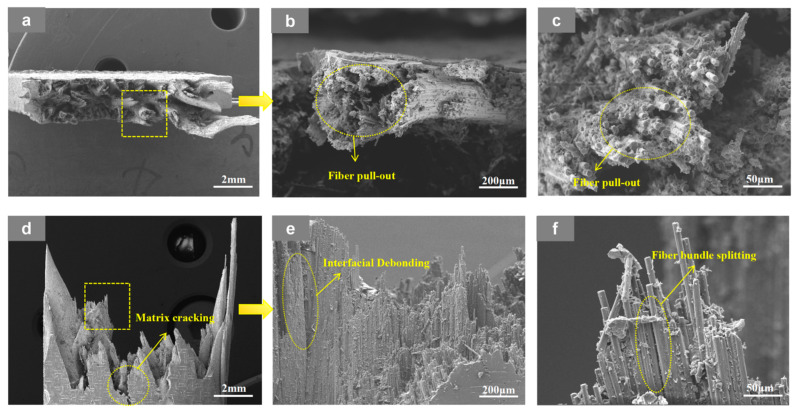
SEM images of the fracture surface of the 3D5A-20° specimen after tensile testing at room temperature (RT), shown at different magnifications: (**a**) top views at 20×; (**b**) top views at 200×; (**c**) top views at 1000×; (**d**) front views at 20×; (**e**) front views at 200×, and (**f**) front views at 1000×.

**Figure 11:**
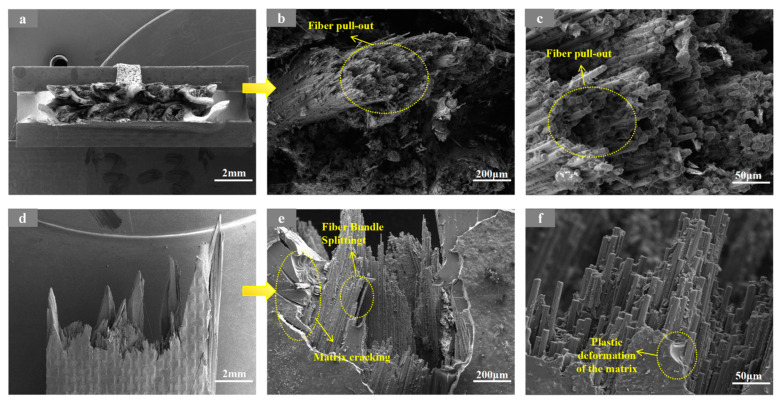
SEM images of the fracture surface of the 3D5A-20° specimen after tensile testing at 90 °C at room temperature (RT), shown at different magnifications: (**a**) top views at 20×; (**b**) top views at 200×; (**c**) top views at 1000×; (**d**) front views at 20×; (**e**) front views at 200×, and (**f**) front views at 1000×.

By comparison, the 3D5A-20° specimens show a more distributed and stable fracture morphology under the same braiding angle. At RT, as shown in Figure 10, the fracture surface is characterized by fiber pull-out, matrix cracking, interfacial debonding, and fiber bundle splitting. Unlike the 3D4A-20° composite, damage does not concentrate as strongly along a single dominant path; instead, the presence of fiber bundle splitting and more dispersed matrix cracking suggests that the additional axial yarn system promotes load sharing and multiple damage paths, which improves energy dissipation and delays localized failure. At 90 °C, as shown in Figure 11, thermal softening increases fiber pull-out, matrix cracking, and local plastic deformation of the matrix, but the fracture morphology still remains relatively distributed, with clear evidence of bundle splitting and multi-region damage development rather than rapid collapse into a single dominant crack path. This indicates that the five-axial architecture retains stronger three-dimensional constraint and stress redistribution at elevated temperature, which helps moderate the acceleration of thermal damage.

Overall, the SEM observations show that both materials undergo more severe interfacial degradation and matrix softening at elevated temperature, but their thermal sensitivities differ markedly because of the internal architecture. The 3D4A-20° composite is more prone to concentrated debonding, rapid crack growth, and earlier unstable failure, whereas the 3D5A-20° composite maintains a more distributed fracture pattern with stronger load sharing and more gradual damage development. Under the same 20° braiding angle, the introduction of the fifth axial yarn therefore improves structural stability at RT and reduces the severity of temperature-induced damage acceleration.

### 3.4. Internal Damage Evolution

**Figure 12:**
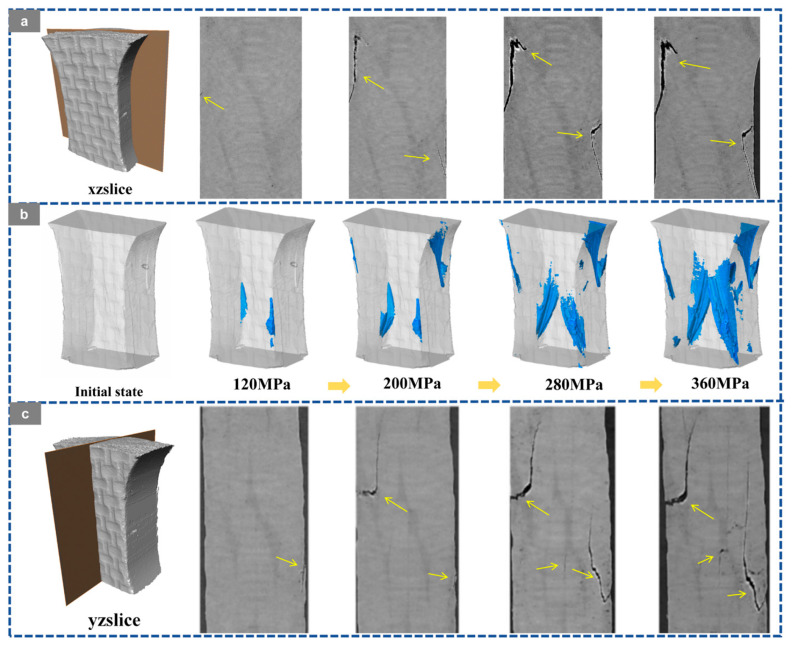
Crack evolution of the 3D braided composites at room temperature (RT): (**a**) XZ CT slices of 3D4A-20°; (**b**) 3D crack morphology of 3D4A-20° at different load levels; (**c**) YZ CT slices of 3D4A-20°.

**Figure 13:**
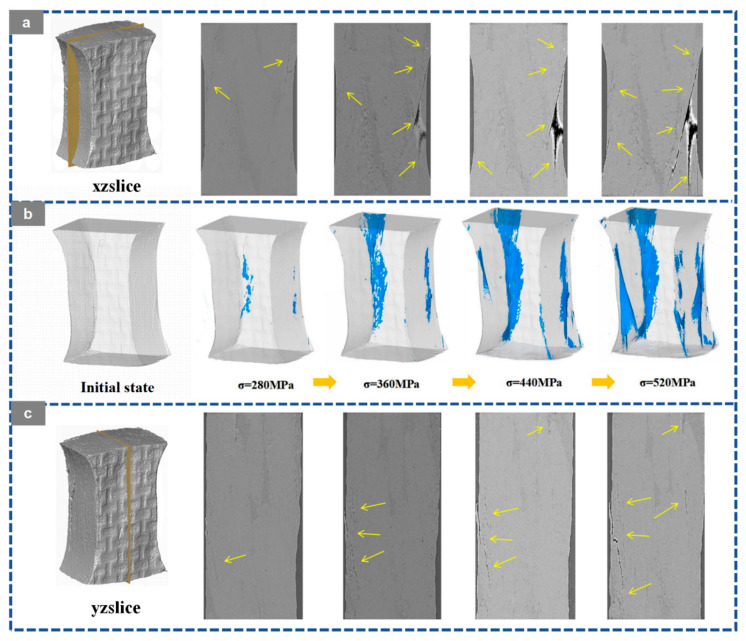
Crack evolution of the 3D braided composites at room temperature (RT): (**a**) XZ CT slices of 3D5A-20°; (**b**) 3D crack morphology of 3D5A-20° at different load levels; (**c**) YZ CT slices of 3D5A-20°.

**Figure 14:**
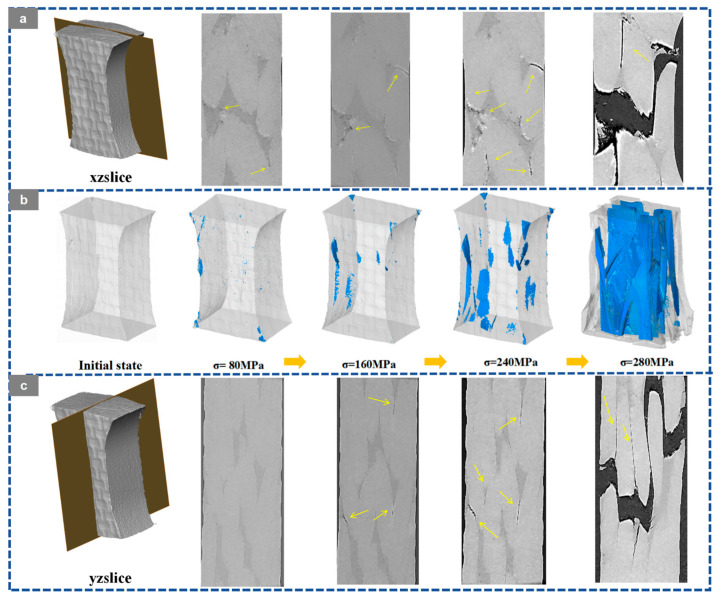
Crack evolution of the 3D braided composites at 90 °C: (**a**) XZ CT slices of 3D4A-20°; (**b**) 3D crack morphology of 3D4A-20° at different load levels; (**c**) YZ CT slices of 3D4A-20°.

**Figure 15:**
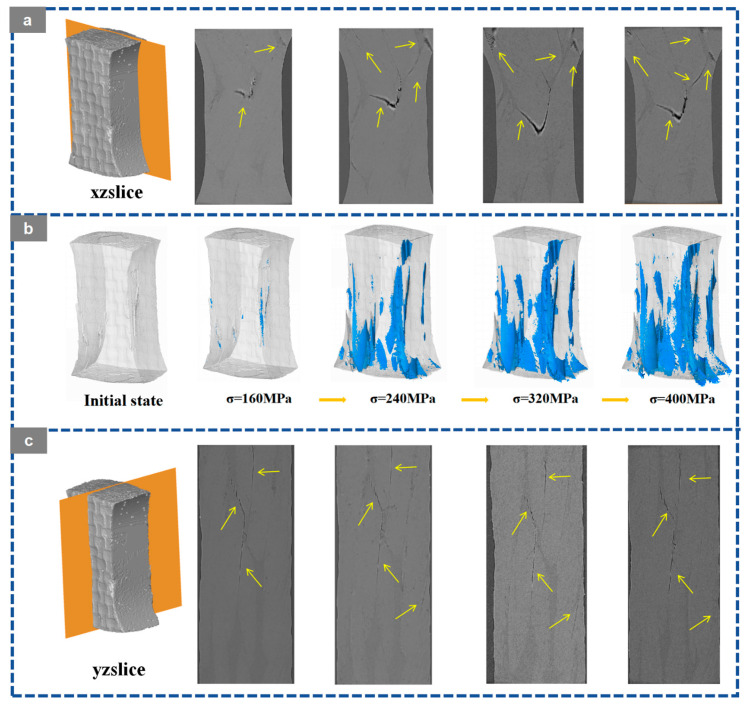
Crack evolution of the 3D braided composites at 90 °C: (**a**) XZ CT slices of 3D5A-20°; (**b**) 3D crack morphology of 3D5A-20° at different load levels; (**c**) YZ CT slices of 3D5A-20°.

**Figure 16:**
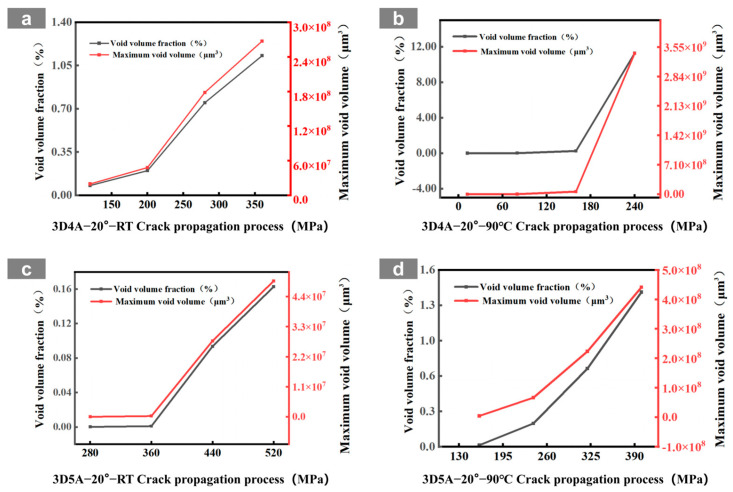
Crack volume evolution in 3D braided composites: (**a**) 3D4A-20° at room temperature (RT); (**b**) 3D4A-20° at 90 °C; (**c**) 3D5A-20° at room temperature (RT); (**d**) 3D5A-20° at 90 °C.

Based on the in situ X-ray CT slices in the XZ and YZ planes, the three-dimensional crack reconstruction, and the corresponding quantitative statistics shown in Figure 12, Figure 13, Figure 14, Figure 15 and Figure 16, the damage evolution of the present 20° braided composites can be interpreted through a temperature-architecture coupling framework. The arrows indicate the crack propagation process. In the current comparison, both specimens have the same braiding angle, while the axial architecture changes from the four-axial system to the five-axial system. Under this condition, temperature mainly governs matrix and interfacial degradation, whereas the axial yarn architecture determines the crack-connectivity mode by modifying load-transfer topology and three-dimensional constraint.

At room temperature, the 3D4A-20° composite exhibits a typical progressive accumulation mode. Microcracks can be identified at about 120 MPa, mainly in yarn interlacing regions and near fiber-matrix interfaces. With increasing load, these cracks grow in a dispersed manner and gradually coalesce along structurally weak paths. As the stress rises from 200 to 360 MPa, the crack network develops from isolated local damage into a more continuous dominant propagation path. The quantitative results are consistent with this transition, with the crack volume fraction increasing from 0.08% to 1.13% and the maximum crack volume rising from the order of 10^7^ to 10^8^ μm^3^. By comparison, the 3D5A-20° composite shows a more delayed and less abrupt crack-evolution process at room temperature. Crack activity remains limited over a broader stress range, and the three-dimensional crack morphology develops more gradually. This behavior indicates that the additional axial yarn system improves load sharing and enhances three-dimensional stress redistribution, thereby suppressing early crack coalescence and delaying the formation of a dominant connected crack path. In other words, under the same 20° braiding angle, the five-axial architecture reduces the tendency for premature crack localization at room temperature.

At 90 °C, the crack-evolution behavior of both architectures changes markedly because matrix softening and interfacial degradation reduce the resistance to crack initiation and growth. For 3D4A-20°, crack nucleation occurs at a lower stress level, and the crack network rapidly develops into a connectivity-dominated failure mode. Once the load approaches the intermediate stage, the reconstructed crack field shows abrupt growth and rapid linkage of previously separated cracks, followed by the formation of a through-thickness connected damage zone. The quantitative data show a sharp increase in crack metrics near the critical load, confirming that thermal degradation promotes unstable crack coalescence in the four-axial structure. In contrast, the 3D5A-20° composite also exhibits earlier crack initiation at elevated temperature, but the subsequent crack evolution remains comparatively more gradual. Cracks develop over multiple regions and expand through several paths rather than collapsing immediately into a single dominant connected band. The gradual increase in crack volume fraction and maximum crack volume indicates that the five-axial architecture still retains a stronger capacity for stress redistribution under thermal loading. As a result, although elevated temperature accelerates overall damage propagation in both materials, the 3D5A-20° composite is less prone to rapid connectivity-driven failure than 3D4A-20°.

Overall, the in situ CT observations and three-dimensional crack reconstruction demonstrate that temperature primarily accelerates internal damage by degrading the matrix and interface, while the axial architecture controls how rapidly local cracks evolve into a connected crack network. Under the same braiding angle, the 3D4A-20° composite is more susceptible to concentrated crack growth and abrupt crack linkage, particularly at elevated temperature. The 3D5A-20° composite, by contrast, maintains a more distributed and progressive crack-evolution process because the additional axial yarns provide higher load-transfer redundancy and stronger three-dimensional constraint. These results confirm that modifying the axial architecture is an effective structural route for improving crack-growth stability and thermal damage tolerance in 3D braided composites.

### 3.5. Internal Strain Evolution Based on DVC

**Figure 17:**
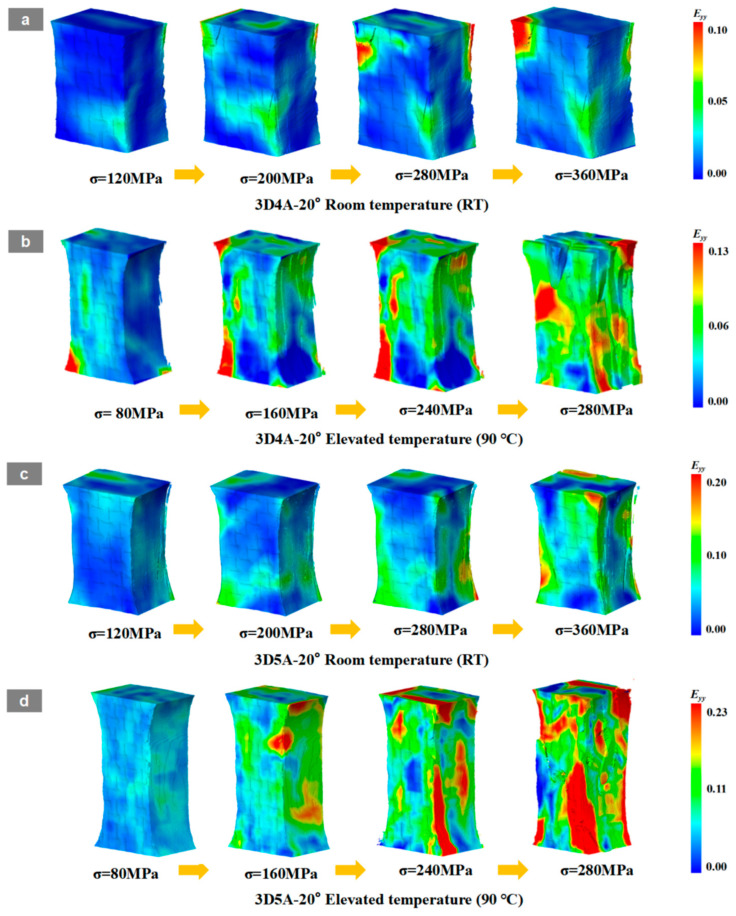
DVC-derived full-field axial strain maps (Eyy) of 3D four-directional braided composites and 3D five-directional braided composites under tensile loading at room temperature (RT) and 90 °C: (**a**) 3D4A-20° at RT; (**b**) 3D4A-20° at 90 °C; (**c**) 3D5A-20° at RT; (**d**) 3D5A-20° at 90 °C.

As shown in Figure 17, the DVC-derived full-field axial strain E_yy_ maps indicate a clear coupling between temperature and axial architecture in 3D braided composites. Temperature accelerates damage by promoting earlier and more intense strain localization through matrix softening and interfacial degradation, while the axial architecture governs the spatial development of localization by modifying load-transfer topology and three-dimensional constraint.

At room temperature, 3D4A-20° exhibits progressive strain concentration along the yarn-aligned load path and develops a dominant fiber-parallel high-strain band at higher stress levels, reflecting load-transfer concentrated in a limited number of aligned yarn channels. In contrast, 3D5A-20° maintains a more stable and spatially distributed strain field over the same loading range, consistent with improved load sharing and enhanced three-dimensional stress redistribution enabled by the additional axial yarn system.

At 90 °C, strain localization initiates earlier and intensifies in both architectures. In 3D4A-20°, high-strain regions appear at lower stress levels and rapidly consolidate into a dominant band, indicating that thermal degradation facilitates the transition from yarn-guided load-transfer to preferential crack linkage. By comparison, 3D5A-20° retains a more distributed localization pattern that develops over multiple regions, demonstrating a greater capacity for strain redistribution at elevated temperature.

Overall, the DVC results confirm that elevated temperature promotes earlier localization in both materials, whereas the five-axial architecture reduces temperature sensitivity by sustaining distributed deformation and delaying the formation of a single dominant localization path. DVC results are in good agreement with the previously observed DIC strain-localization patterns, crack-evolution behavior, and fracture-morphology analysis, further confirming the consistency of the multi-scale damage characterization in the present study.

## 4. Conclusions

This work clarifies the thermal-mechanical tensile response and multi-scale damage evolution of low-braiding-angle 3D braided CFRP composites with two axial architectures, 3D4A-20° and 3D5A-20°, by integrating in situ X-ray CT, full-field DIC and DVC, and SEM fractography.

(1) Architecture controls both baseline performance and the dominant damage topology across scales. At room temperature, 3D5A-20° shows higher tensile modulus and strength than 3D4A-20°. The multi-scale observations consistently indicate that the additional axial yarn system strengthens three-dimensional constraints and load sharing, so deformation and damage remain more distributed and the transition to a single dominant crack path is delayed.

(2) Elevated temperature is the primary driver of damage acceleration through matrix softening and interfacial weakening. At 90 °C, both architectures exhibit earlier localization, earlier crack initiation, and faster internal crack growth. The macroscopic response degrades accordingly, with 3D4A-20° showing larger reductions in modulus and strength (28.20% and 16.31%) compared with 3D5A-20° (2.42% and 10.25%).

(3) Axial architecture regulates temperature sensitivity by governing crack connectivity. Under thermal exposure, 3D4A-20° is more prone to concentrated localization and rapid crack linkage once the matrix and interface lose constraint, which promotes the earlier development of connected cracking. In contrast, 3D5A-20° maintains a more progressive damage evolution with multi-path crack development, reflecting more effective stress redistribution and improved resistance to early connectivity-driven failure.

(4) The 3D5A architecture is more suitable for blade-like structures subjected to tensile-dominated service loads. Owing to its consistently higher tensile modulus and strength, stronger load-transfer redundancy, and better resistance to temperature-induced damage acceleration, 3D5A-20° provides a more favorable structural configuration for blade applications in which tensile load-carrying capability and damage tolerance are critical.

Overall, introducing the fifth axial yarn provides a practical architecture route to improve load-transfer redundancy and enhance thermo-mechanical damage tolerance for low-braiding-angle 3D braided composites in elevated temperature service. Future work will focus on the thermo-mechanical damage evolution of low-braiding-angle 3D braided CFRP composites under more realistic service conditions, such as fatigue, impact, and coupled thermo-mechanical cyclic loading. In addition, extending the present multi-scale characterization approach to full-scale blade-like structures will provide further guidance for structural optimization and engineering applications at elevated temperatures.

## Figures and Tables

**Table 1:** Structural parameters of 3D braided composites.

Properties	3D4A-20°	3D5A-20°
Fiber volume fraction (%)	53.9	55.8
Fiber content (g)	426	426
Actual braiding angle (°)	20.1	20.2

**Table 2:** Mechanical properties of different materials.

Materials	Tensile Modulus at RT	Tensile Strength at RT	Tensile Modulus 90 °C	Tensile Strength at 90 °C
Resin matrix	2.12 GPa	82.44 MPa	1.70 GPa	34.99 MPa
T300-3K	230.00 GPa	3530.00 MPa	230.00 GPa	3530.00 MPa

**Table 3:** Mechanical properties of woven composites with different braiding architectures.

Properties	3D4A-20°		3D5A-20°	
	Tensile Modulus/GPa	Tensile Strength/MPa	Tensile Modulus/GPa	Tensile Strength/MPa
RT	76.6 ± 1.8	617.6 ± 7.8	89.5 ± 2.1	807.1 ± 18.4
90 °C	55.0 ± 2.9	516.9 ± 8.6	79.3 ± 2.1	778.9 ± 20.8
Reduction (%)	28.20	16.31	2.42	10.25

## Data Availability

The original contributions presented in this study are included in the article. Further inquiries can be directed to the corresponding authors.

## Abbreviations

CFRP	carbon-fiber-reinforced polymer
3D4A	3D four-directional
3D5A	3D five-directional
CT	computed tomography
DIC	digital image correlation
DVC	digital volume correlation
